# Crosstalk Between Acid Sphingomyelinase and Inflammasome Signaling and Their Emerging Roles in Tissue Injury and Fibrosis

**DOI:** 10.3389/fcell.2019.00378

**Published:** 2020-01-14

**Authors:** Cao Li, Shanshan Guo, Wenyuan Pang, Zhigang Zhao

**Affiliations:** ^1^Department of Pharmacy, Beijing Tiantan Hospital, Capital Medical University, Beijing, China; ^2^Department of Clinical Pharmacology, School of Pharmaceutical Sciences, Capital Medical University, Beijing, China

**Keywords:** acid sphingomyelinase, ceramide, inflammasome, fibrosis, lysosome, ROS, NLRP3, caspase-1

## Abstract

Inflammasomes are a group of protein complexes that are assembled by pattern recognition receptors following the recognition of invading pathogens or host-derived danger signals. Inflammasomes such as NLRP3 mediate the activation of caspase-1 and the production of the proinflammatory cytokines IL-18 and IL-1β. Regulation of inflammasome signaling is critical for host defense against infections and maintenance of cellular homeostasis upon exposure to multiple harmful stimuli. Recent studies have highlighted an important role of acid sphingomyelinase (ASM) in regulating inflammasome activation. ASM hydrolyzes sphingomyelin to ceramide, which further fuses to large ceramide-enriched platforms functioning in stabilizing and amplifying molecules and receptors. Here, we will discuss the current understanding of the ASM-ceramide system in inflammasome activation, and how it contributes to multiple diseases. Insights into such mechanisms would pave the way for further exploration of novel diagnostic, preventative, and therapeutic targets against tissue injury and fibrosis.

## Introduction

Inflammasomes are composed of a variety of protein complexes located in the cytosol and trigger dimerization of protease caspase-1 and the generation of proinflammatory cytokines IL-18 and IL-1β (Martinon et al., 2002). Activation of different germline-encoded pattern-recognition receptors (PRRs) by pathogen-associated molecular patterns (PAMPs) derived from infectious pathogens or endogenous danger-associated molecular patterns (DAMPs) initiates the assembly of multiple distinct inflammasomes (Guo et al., 2015; Broz and Dixit, 2016). Inflammasomes are important in host defense against invading pathogens; while dysregulated inflammasome signaling is associated with tumors, autoinflammatory, neurologic, and endocrine diseases. Thus, the precise mechanisms of inflammasome activation is critical for the host to fight against infections while avoiding overt tissue damage (Schnappauf et al., 2019).

Emerging studies have revealed that acid sphingomyelinase (ASM) plays crucial role in the activation of inflammasomes (Grassmé et al., 2014; Boini et al., 2016; Koka et al., 2017). ASM is an enzyme that functions to catalyze sphingomyelin into ceramide (Gatt, 1963). Sphingomyelin was characterized as the most abundant sphingolipid and predominantly presents in the outer leaflet of the cell membrane (Slotte and Ramstedt, 2007). Upon exposure to diverse stimuli, ASM catalyzed ceramide drastically changes physiological characteristics of membrane structure, thereby re-organizing molecules and signaling cascades within the cell, including cellular differentiation and proliferation, tumor presentation, pathogen recognition, as well as inflammation signaling pathways (Henry et al., 2013). Here, we comprehensively review recent studies involving ASM-inflammasome, regulatory mechanisms of ASM in inflammasome activation, and their functions in host defense and diseases.

## A Glimpse Inside Inflammasomes

In response to PAMPs or DAMPs, the inflammasome activates caspase-1 and the downstream cytokine IL-1β (Rathinam and Fitzgerald, 2016; Evavold and Kagan, 2019). PAMPs are conserved molecules of microbes, including viruses, bacteria, and fungi, bacterial secretion systems and their effector proteins, while DAMPs are a series of host-derived signals such as ATP, uric acid crystals, reactive oxygen species (ROS), and the heat-shock proteins 90. Specific PRRs trigger the sensor activation, oligomerization, and recruitment of an adaptor protein called apoptosis-associated speck-like protein containing CARD (ASC), which includes a pyrin domain and a caspase recruitment domain (Srinivasula et al., 2002). According to their subcellular localization, PRRs can be divided into two major types: transmembrane proteins found in endosome and the plasma membrane such as Toll-like receptors (TLRs), and intracellular compartments including AIM2-like receptor (ALRs), Nod-like receptors (NLRs), and RIG-I-like receptors (RLRs) proteins (Takeuchi and Akira, 2010; Lamkanfi and Dixit, 2014). ASC is crucial for connecting the upstream inflammasome sensor molecule to caspase-1. Inactive pro-IL-18 and pro-IL-1β are cleaved by protease caspase-1 to generate bioactive inflammatory cytokines IL-18 and IL-1β, respectively, (Srinivasula et al., 2002; Kayagaki et al., 2011). Activation of caspase-1 is also essential for an inflammatory cell death named pyroptosis (Shi et al., 2015).

Advances in the inflammasome activation signaling have been extensively reviewed in detail previously (Broz and Dixit, 2016; Rathinam and Fitzgerald, 2016; Evavold and Kagan, 2019). Here, we briefly give an overview of recent developments in NLRP3 inflammasome, which is the best-characterized inflammasome. Activation of the NLRP3 inflammasome requires two steps: priming and then activating. Basal expression of NLRP3 is thought to be insufficient for NLRP3 activation in resting cells (Bauernfeind et al., 2009). Engagement of PAMPs or DAMPs with PRRs, such as TLRs or Nucleotide-binding oligomerization domain-containing protein 2 (NOD2), leads to nuclear factor- κB (NF-κB) activation and NLRP3 gene transcription (He et al., 2016; Awad et al., 2018; Swanson et al., 2019). Also, interleukin-1 receptor-associated kinases 1 (IRAK1), as well as ubiquitin modifications, play an important role in post-transcriptional regulation in the priming step (Bauernfeind et al., 2009; Juliana et al., 2012). Following the priming of NLRP3, the inflammasome is fully activated. NLRP3 responds to a variety of stimuli signals, such as efflux of K^+^, flux of Ca^2+^, lysosomal rupture, mitochondrial dysfunction, and ROS. For example, cytosolic ROS and ATP activated mitochondrial ROS were demonstrated to be necessary for activation of NLRP3 (Cruz et al., 2007; Dostert et al., 2008). Consistently, inflammasome activation is suppressed when ROS are inhibited (Gross et al., 2016). However, evidence shows that cells lacking nicotinamide adenine dinucleotide phosphate (NADPH) oxidase activity are capable of activating the NLRP3 inflammasome (van Bruggen et al., 2010). The role of ROS in activation of NLRP3 inflammasome is still under debate.

Although the past two decades has witnessed a rapid development of our understanding in inflammasomes, the exact molecular mechanisms of how inflammasomes sense cellular stress and get activated remain to be fully elucidated. The recent advancement of the involvement of ASM in inflammasome signaling may shed light on aspects of physiology and pathology not yet known.

## Acid Sphingomyelinase and ASM-Generated Ceramide

Hydrolysis of sphingomyelin into ceramide and phosphorylcholine plays a central role in the sphingolipids metabolic pathway (Xiong et al., 2016). Reduction or absence of ASM activity leads to deposition of sphingomyelin and lysosomal storage disorders, Niemann-Pick disease types A and B (McGovern et al., 2006; Schuchman, 2007). Initially, ASM was considered only affecting lysosome at an acidic pH (Fowler, 1969), later results show that ASM catalyzes the breakdown of lipoprotein-sphingomyelin even at pH 7.4 (Schissel et al., 1998). This difference in pH reflects the fact that the ASM gene generates two distinct forms of the proteins: secretory ASM (S-ASM) found extracellular and lysosomal ASM (L-ASM) locating in the endo-lysosomal vesicles. Two forms of ASM are resulted from alternative trafficking and posttranslational modification and trafficking of the encoded protein (Ni and Morales, 2006; Wahe et al., 2010; Vazquez et al., 2016). Trafficking of ASM to lysosome is required for clustering of lipid raft in endothelial cells and their function (Jin et al., 2008). Additional studies are required to determine whether these two forms of ASM catalyze different sphingomyelin sources, and how they mediate diverse signaling pathways.

The molecular mechanisms involved in the regulation of ASM are only partially characterized. Multiple stimuli are known for regulating ASM activity, such as direct oxidation, bacterial and viral infection, irradiation, ions treatment, protease, and ROS. Among these, the connection of ROS and ASM is one of the well-investigated (Jin and Zhou, 2009; Li et al., 2010; Jin et al., 2011); for example, activation and extracellular secretion of ASM extracellular can be induced by hydrogen peroxide in various cells (Zhang et al., 2008; Li et al., 2012; Manago et al., 2015). However, the inhibition of ROS by different ROS scavengers, and the fact that ASM is inhibited by the NADPH-oxidase pharmacological inhibitor diphenyleneiodonium chloride (DPI) suggest differences in its regulation (Lang et al., 2007; Zhang et al., 2008; Peng et al., 2015). ASM activation triggered ceramide enriched domains can be abolished by gene silencing of gp91^*phox*^, a subunit of NADPH oxidase (Boini et al., 2010). ASM activation can also be achieved through dimerization by direct oxidation of C-terminal cysteine (Qiu et al., 2003). The above data suggest that ROS are needed for ASM performing its function, however, the accurate mechanism of how ROS direct or indirect control the activation of ASM remains further investigation.

Ceramide is found mainly existing within the plasma membrane (Castro et al., 2014). A low concentration of 5–10 mol% ceramide is adequate to automatically promote the construction of ceramide-rich platforms in phospholipid bilayer (Veiga et al., 1999). ASM cleaves sphingomyelin to ceramide, a lipid known to spontaneously self-associate and to construct small ceramide-rich membrane rafts. The fusing of these membrane rafts builds large ceramide-rich platforms, which serve to signal transitions (Zhang et al., 2009; Gulbins et al., 2015).

In general, the accumulation of ceramide and the construction of ceramide-rich platforms appear to facilitate the transport membrane proteins, aggregate cellular receptors, deliver signal molecules after exposure to diverse stimulations. Ceramide participates in multiple physiological and pathological activities. First, as previously described, the acyl chain of ceramides interacts with each other there by fix the lipids rafts tightly (Kolesnick et al., 2000), which further generates the structure of ceramide-enriched domains to a stable lipid gel state. Second, these domains reorganize and cluster receptors and signaling molecules, such as CD95 (Grassmé et al., 2001), CD20 (Bezombes et al., 2004), death receptor 5, and CD40 (Grassmé et al., 2002). Clustering of these molecules by ceramide may leads to a large increase in receptor density, thus structural modifications of the receptors, activation of the downstream signaling targets, and excluding of suppressive proteins. Furthermore, ceramide can serve as a secondary messenger. Ceramide domains can interact and activate various enzymes, for example, lysosomal hydrolase cathepsin D (Zebrakovska et al., 2011), phospholipase A2 (Bharath et al., 2015), and kinase suppressor of Ras (Zhang et al., 2011). Recent studies indicate that the ASM-ceramide system is critical for the host to recognize, internalize, and eliminate infectious microorganisms (Li et al., 2019). ASM, ceramide, and ceramide platforms are essential for reorganizing cellular signalosome complex, thereby permitting stressful stimulations and transduction of biological signals to regulate cells and tissues.

## Interaction Between ASM and the Inflammasome

The ASM-ceramide system functions in a variety of physiological processes, such as cell death, proliferation, growth, and differentiation. The ASM gene is expressed ubiquitously in plasma membrane and organelles which suggests its significance in maintenance of regular membrane turnover and remodeling; therefore, the involvement of ASM-ceramide system would be a reasonable mechanism in regulating inflammasome signaling.

### ASM Links PAMPs With the Inflammasome

Recent studies demonstrate a signaling cascade from ASM derived ceramide via the inflammasome to caspase-1 and the release of IL-1β and IL-8 (Bianco et al., 2009; Cuzzocrea et al., 2009; Grassmé et al., 2014), which strongly indicates that this enzyme is linked with inflammation signaling. However, none of the above studies investigated precise molecular machinery of how the ASM-ceramide system regulates inflammasome signaling. The author conducted the investigation using cystic fibrosis transmembrane conductance regulator (CFTR) gene deficient mice and human cystic fibrosis (CF) lung samples (Grassmé et al., 2014). The study showed that the accumulation of ceramide results in caspase-1 protein level upregulation and translocation of the protein to the luminal membrane in epithelium of CF mice; moreover, ASC expression is increases in the lungs of CFTR-deficient mice. Both the dysregulation of ASC and caspase-1 are corrected by heterozygosity of the ASM gene. The novel concept that ceramide accumulation leads to inflammasome activation is consistent with a previous finding that, ceramide triggers activation of the inflammasome and subsequently generation and secretion of proinflammatory cytokines as well as the permeability of alveolar epithelial cells (Kolliputi et al., 2012). ASM-generated ceramide-enriched membrane domains may directly induce recruitment and cluster of ASC to initiate the activation of the inflammasome similarly to CD95 or CD40. The association of ASC and ceramide may lead to the downstream activation of caspase-1. The ASM-ceramide system may also act in an indirect way to regulate the inflammasome.

Recently, a further study investigated the role of ASM in inflammasome signaling (Ma et al., 2017). *Staphylococcus aureus* alpha-toxin (α-toxin) is a pore-forming toxin that triggers membrane permeabilization and induces inflammation (Cohen et al., 2016). In an *ex vivo* macrophages infection model, α-toxin stimulation resulted in the sustained ASM activation and the release of ceramide locating in lysosomes, this effect is absent in ASM deficient cells. The release of lysosomal ceramide upon exposure of cells to α-toxin stimuli induces permeabilization of the lysosome, and trafficking of lysosomal hydrolase cathepsin D and B to the cytosol. Interestingly, although both cathepsin D and B are released into the cytoplasm, confocal microscopy and co-immunoprecipitation experiments show that only cathepsin B associates with ASC and the inflammasome protein NLR family CARD domain-containing protein 4 (Nlrc4), and thereby induces the release of IL-1β. All these effects are prevented or attenuated by knockout of the ASM gene or pretreatment with its pharmacological inhibitor amitriptyline, suggesting the importance of ASM in the induction of proinflammatory cytokines in macrophages given a *S. aureus* α-toxin challenge. Ceramide has previously been shown to induce lysosomal activation of cathepsin B, which is related to endoplasmic reticulum stress, autophagy, and apoptosis (Taniguchi et al., 2015; Liu et al., 2016). There might be two possible mechanisms involving ASM and α-toxin induced cathepsin B activation: first, ceramide can directly interact with cathepsin; second, fusion of *S. aureus* contained phagosomes with acidified lysosomes (Li et al., 2017) possibly makes α-toxin induce lysosome permeabilization and promote the translocation of cathepsins. Notably, leakage of LPS from bacteria invaded phagolysosomes or cytosolic gram-negative bacteria may activate a non-canonical NLRP3 inflammasome (Kayagaki et al., 2013).

Release of cathepsins caused by lysosomal rupture is crucial for inflammasome activation (Hornung et al., 2008; Liu et al., 2016). The pharmacological inhibitor CA-074Me prevents the inflammasome signaling activating and the IL-1β production upon exposure to α-toxin (Ma et al., 2017). Besides, the mature form of cathepsin D follows the ceramide production pattern (Spengler et al., 2018). A bacterial extract of *Lactobacillus casei* cell wall fragments (LCWE), induces the formation of the NLRP3 inflammasome and colocalization of NLRP3 with ASC or caspase-1. This is dependent on cathepsin B activation, which is blocked by ASM siRNA, or disruption of ceramide membrane rafts disruption (Chen et al., 2016b). These results prove that the ASM-ceramide system induces NLRP3 inflammasome activation through the lysosome-cathepsin B pathway. However, activation of NLPR3 inflammasome shows a moderate or even no defectiveness in cathepsin B deficiency cells (Dostert et al., 2009). A possible mechanism would be that pharmacological cathepsin B inhibitor could exert off-target or suppress other cathepsins. Further, small numbers of studies show that cathepsin B triggers the inflammasomes activating upon toxins stimulation (Ali et al., 2011; Gupta et al., 2014). The mechanism linking ASM regulated lysosomal permeabilization to inflammasome remains to be determined.

Based on the above studies, ASM generated ceramide may either directly associate with the inflammasome, or indirectly interact with NLRP3 by regulating the lysosomal activation of cathepsin B or D. The ASM-ceramide system would, thus, be a promising therapeutic target treating lung injury.

### ASM Links DAMPs With the Inflammasome

Recent study demonstrated that ASM and ceramide contribute to NLRP3 inflammasome formation and activation in hypercholesterolemia mice (Koka et al., 2017). 7-ketocholesterol or cholesterol crystals dramatically induced the formation and activation of NLRP3 inflammasomes, clustering of NLRP3-ASC-caspase-1 complex, activation of caspase-1, and production of IL-1β, these events were dramatically suppressed by gene silence of ASM, pharmacological inhibition, or gene deficiency in mice carotid arterial endothelial cells (CAECs). Similar results of increased ASM expression, enhanced ceramide production, and inflammasome complex formation are observed in the carotid arteries *in vivo*. Moreover, endothelial dysfuction is attenuated by NLRP3 gene deficiency in hypercholesterolemia mice (Zhang et al., 2015). These findings are in consistent with previous results by the same group that ASM gene deficiency reduces activation of NLRP3 inflammasome and alleviates obesity-induced glomerular injury from western-diet (Boini et al., 2016). Further, the study showed that the NOX subunits gp91^*phox*^ is aggregated in membrane rafts upon 7-ketocholesterol or cholesterol crystal stimulation, which are abolished by ROS scavenger or genetic silence of thioredoxin interacting protein (a ROS-dependent activator of NLRP3). In addition, multiple stimuli including homocysteine or visfatin, induce activation of NOX-derived ROS by formation of membrane rafts redox signalosomes (Abais et al., 2013; Xia et al., 2014). These studies strongly support the idea that NOX subunits cluster on membrane rafts and produce O^2–^, which can further trigger downstream inflammasome activation. Although ROS activating NLRP3 inflammasome complex is a commonly adopted mechanism, a topic of longstanding debate still exists about the role of ROS in NLRP3 activation (Lawlor and Vince, 2014; Elliott and Sutterwala, 2015). Dysfunction of NADPH oxidase in mouse macrophages or human mononuclear cells still has a regular NLRP3 activation (van Bruggen et al., 2010). Chemical inhibitors are likely to cause off-target artifacts; a study shows that a high concentration of ROS inhibitors DPI or NAC affects the NLRP3 inflammasome in the priming stage (Bauernfeind et al., 2011). Thus, additional studies are required to evaluate the role of ROS in NLRP3 activation.

The ASM-ROS-NLRP3 inflammasome axis plays a crucial role in barrier properties of endothelial cells (Peng et al., 2015; Koka et al., 2017). Formation of redox signaling platforms and clustering of lipid rafts regulate signaling of death receptors Fas-ligand (Zhang et al., 2006) and TRAIL-death receptor 4 (Li et al., 2013), inducing dysfunction of endothelial cells. The use of ASM chemical inhibitor amitriptyline, ASM gene siRNA, or genetic deficiency significantly decreases activated inflammasome complex and reduces endothelia cell permeability. The inhibition of this axis acts as a non-canonical NLRP3 inflammasome pathway, which may protect endothelium function as well as prevent hypercholesterolemia induced atherogenesis (Koka et al., 2017); interestingly, NLRP3 inflammasome strikingly increase susceptibility of macrophages and migration upon lipid stimulation, which may finally promote atherosclerosis (Li et al., 2014). Furthermore, a study reveals that ASM promotes NLRP3 inflammasome activation induced by membrane injury in endothelial cells (Chen et al., 2016b). Formation and activation of NLRP3 inflammasomes is mediated by membrane raft clustering, which impairs plasma membrane resealing. Fail of the membrane resealing process leads to lysosome rupture, release of cathepsin B, and subsequently activation of NLRP3 inflammasomes upon LCWE stimulation, which are abolished by ASM gene silencing or inhibition of membrane raft clustering. Additionally, NLRP3 gene deficiency inhibits degradation of tight junctions in mice endothelial cells during high-fat diet induced vascular injury *in vivo* (Chen et al., 2015, 2016a; Wang et al., 2016).

Taken together, these finding shows that modulation of the ASM-ceramide system may protect endothelial cells or artery function from pathological changes resulting from inflammasome activation.

### The ASM-Inflammasome Interaction in Tissue Injury and Fibrosis

#### Cystic Fibrosis and Pulmonary Injury

Cystic fibrosis is a genetic autosomal recessive disease commonly seen in Caucasians and is resulted from mutations in CFTR gene, which leads to chronic inflammation and recurrent and chronic bacterial infections (Cantin, 2019). Multiple mechanisms have revealed that ASM regulates inflammation signaling. Marked accumulation of ceramide and elevated ASM activity increase epithelial cell death and IL-8 production in CF mice (Becker et al., 2010; Grassmé et al., 2014). Long-term (6.5 months) treatment with pharmacological inhibitors of ASM or genetic heterozygosity minimizes pulmonary inflammatory cytokines and significantly decreases the development of lung fibrosis in CF mouse (Ziobro et al., 2013). Moreover, systematic applying of The Food and Drug Administration (FDA) approved medicine amitriptyline strongly benefits pulmonary function and infection vulnerability in CF patients (Nahrlich et al., 2013). Further studies show direct evidence that ASM induces activation of the inflammasome complex by membrane recruiting of ASC and caspase-1 (Koka et al., 2017).

Acid sphingomyelinase-ceramide system acts as key players in a piglet acute respiratory distress syndrome (ARDS) model (Spengler et al., 2018). Inositol 1,2,6-trisphosphate (IP3) and phosphatidylinositol 3,5-bisphosphate (PIP2) significantly reduce pulmonary edema and increase the oxygenation index as well as ventilation index by suppressing ASM and ASM-dependent ceramide production, which are further connected to inhibition of NLRP3-ASC-caspase-1 axis. The subsequent decreased production of IL-1β and TNF-α by PIP2 and IP3 reduces consecutive ceramide-induced apoptosis and maintain permeability in alveolar epithelial cells. This result is consistent with a previous study, which showed ASM inhibition attenuates lysosomal cathepsin D activation (Kolliputi et al., 2012). The NF-κB pathway is activated although its interaction with ASM is not yet determined. ASM inhibition decreases the protein level of transforming growth factor-β1 (TGF-β1) and IFN-γ, which are strongly correlated with a reduction in elastin and MMP-1 expression. The inhibition of TGF-β1 is an effective way to alleviate lung fibrosis (Yamaguchi et al., 2012).

#### Renal Fibrosis

The homeostasis of ASM-ROS-NLRP3 inflammasome axis is required for the maintenance of endothelial cell integrity hypercholesterolemia-induced in renal fibrosis (Han et al., 2018). TGF-β1 induces the membrane raft formation and ROS production pathways in renal tubular cells, which are attenuated by ASM gene silencing associated epithelial–mesenchymal transition (EMT). Moreover, extracellular signal-regulated kinase 1 (Erk) 1/2 activation may act as a downstream regulator of the membrane rafts derived ROS. *In vivo* data show that the renal tubular membrane rafts-redox signaling pathway is activated, and the endothelial cells permeability is markedly increased in angiotensin II-induced hypertension, these changes are significantly reduced by pharmacological inhibition, siRNA, or gene deficiency. These findings are consistent with a previous study showing that ASM inhibition improved renal function and fibrosis in an animal model (Achar et al., 2009). Notably, inhibition of the ASM signaling pathway decreases TGF-β1 induced EMT, one of the major mechanisms of tubulointerstitial fibrosis.

Hong et al. (2019) recently showed that ceramide regulates lysosome function through activation of NLRP3 inflammasome and induction of production of IL-1β via extracellular vesicles upon exposure of D-ribose stimulation in podocytes. Both endogenous derived and exogenous administrated D-ribose induces the activation of NLRP3 and secretion of IL-1β, which further lead podocyte injury, chronic sterile glomerular inflammation, and glomerular sclerosis. Ceramide is required for extracellular vesicles formation, secretion, and function such as delivery of effector molecules (Elsherbini and Bieberich, 2018). Inhibition of lysosomal ceramide decreases extracellular vesicle secretion, thereby reducing the transport of NLRP3 inflammatory cytokines from the podocytes to exercise their function in initiating glomerular injury and renal fibrosis.

#### Silicosis

In a mouse model, the ASM inhibitor imipramine blocked acute silicosis, which is one of the most prevalent diseases as a result of occupational exposures (Biswas et al., 2017). Imipramine significantly attenuates cytotoxicity and production of IL-1β in silica stimulated alveolar macrophages *in vitro*. Furthermore, *in vivo* data shows that imipramine alleviated lung injury and collagen accumulation in a short or long-term silica-induced mouse model. The underlying mechanism is that imipramine significantly lowers silica-induced phagolysosomes permeabilization and the subsequent lung silicosis. Further investigations are required to characterize the action of imipramine and ASM inhibition in the context of particle-induced inflammation.

Acid sphingomyelinase regulated lysosome function in silicosis may be associated with macrophage receptor with collagenous structure (MARCO), a scavenger receptor expressed in tissue resident macrophages for recognizing and clearing of pathogens and environmental particles such as silica expressed (Biswas et al., 2014). In MARCO^–/–^ macrophages, silica exposure induced ASM activation and accumulation of ceramide mediates lysosomal membrane permeabilization and cathepsin B releases, which further leads to the formation of the NLRP3-caspase-1 complex as well as IL-1β release. This study supports the concept that ceramide level increases and sphingomyelin level decrease in MARCO deficiency upon silica exposure (Johansson et al., 2010).

#### Liver Injury and Fibrosis

The role of ASM in liver injury is still under debate. In a bile duct ligation induced mice liver injury model, protein level of ASM and ceramide concentration increased, in addition, chimeric mice containing ASM^–/–^ bone marrow cells showed an increased TNF-α and IL-1β production after ligation. The study finds that ASM and ceramide are required for Kupffer cell function in reducing inflammation, inducing hepatocyte survival and regeneration, and preventing liver fibrosis (Osawa et al., 2010). In contrast, another study shows that combined IL-6 and ASM inhibition significantly reduces hepatocyte apoptosis, cytokine and chemokine production, and liver fibrosis in bile duct ligated mice (Hubel et al., 2017). In addition, recent studies show that inhibition of ASM exhibited a protective effect on liver function. Long term sepsis survivor develops liver fibrosis from hepatic stellate cell (HSC) activation (Gonnert et al., 2012), and heterozygote expression of ASM or pharmacological treatment with desipramine improves liver function and fibrosis by decreasing the production of cytokine IL-1β and MCP1 (Chung et al., 2017). The controversial action of ASM on liver fibrosis may be due to the various functions of ceramide, the use of different cells, or different murine models in different studies.

#### Brain Injury

Acid sphingomyelinase has a fundamental role in mitochondrial dysfunction promoted neuroinflammation and secondary brain injury (Novgorodov et al., 2019). ASM is activated without a change of protein level via post-transcriptional mechanisms in response to traumatic brain injury, which might be due to dimerization of the enzyme (Qiu et al., 2003). ASM is required for traumatic brain injury-induced mitochondrial respiratory chain dysfunction, which leads to enhancement of oxidative protein modification in the injured brain. Interestingly, a previous study showed that the mitochondrial respiratory chain induces activation of mitochondrial ASM and formation of ceramide (Manago et al., 2015), these data may indicate a positive activation feedback mechanism of mitochondrial ROS and ASM.

In addition, brain injury triggers increasing in NLRP3 and caspase-1 expression, which is significantly attenuated in ASM-deficient or pharmacologically inhibited mice (Novgorodov et al., 2019). This indicates that ASM-ceramide system is an essential factor for assembling and activating the NLRP3 inflammasome complex in neurological inflammatory cascade. Of note, this study suggests that ASM is a required factor promoting astrocyte activation in reactive astrogliosis development during the brain response against trauma, thus, ASM-ceramide system would be a favorable target for the development of clinical treatment to brain trauma injury.

In summary, the ASM-ceramide system is uniquely positioned in activating multiple signaling cascades, including lysosomal cathepsin release, TGF-β1 expression, and ROS production. These molecules further activate the NLRP3 complex, induce the release of proinflammatory cytokines, and lead to tissue injury and fibrosis.

## Conclusion and Future Perspectives

Despite multiple studies showing the role of ASM in inflammasome signaling, the identification of mechanisms involved in the control of ASM function in health and disease will provide important information on how such a ubiquitously expressed gene is regulated. This will hopefully give us a novel detailed paradigm of the molecular basis of inflammasome signaling. Ultimately, this could pave the way for the discovery of novel therapeutic targets for tissue injury and fibrosis.

**FIGURE 1:**
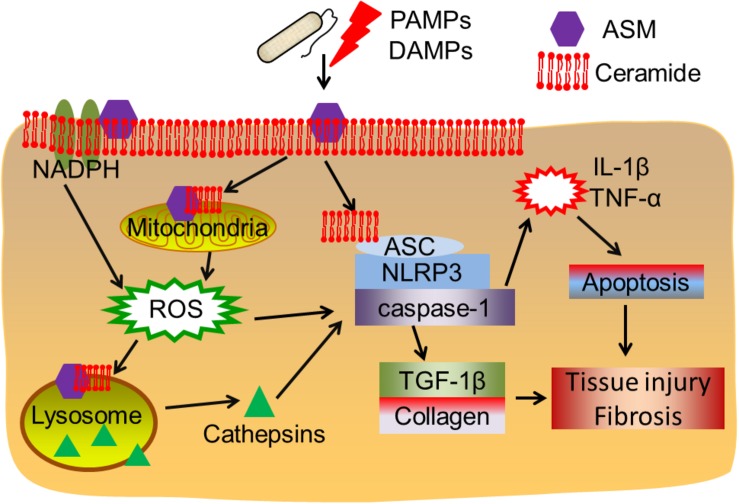
Acid sphingomyelinase/ceramide system in inflammasome signaling and tissue fibrosis. Upon PAMPs or DAMPs exposure, ASM is activated and ceramide enriched domains are formed. Ceramide enriched domains may either directly interact with the ASC-NLRP3-caspase-1 inflammasome complex, or, ASM-ceramide system may induce NADPH oxidase or mitochondrial ROS production or cathepsins released after lysosome rupture, leading to inflammasome activation. The subsequent cytokine production and TGF-β1 activation may contribute to cell apoptosis, tissue injury and fibrosis.

Overall, the findings discussed in this review highlight that ASM-ceramide system regulated signalings (Figure 1), particularly lysosomal function and ROS production are associated with inflammasome activation. As shown in the earlier part of this review, upon PAMPs (bacteria, LPS, and toxins) or DAMPs (hypercholesterolemia, cholesterol crystal, D-ribose, silica etc.) exposure, ceramide enriched membrane platforms are constructed according to ASM activation and ceramide generation. Subsequently, ceramide may directly cluster and condense ASC and the NLRP3 inflammasome, this will further lead to activation of caspase-1, proinflammatory cytokines release, and tissue injury. However, the ASM-ceramide system may regulate the inflammasome complex in an indirect manner: (1) ASM induced ceramide production results in lysosome rupture and the secretion of lysosomal cathepsin B or D to cytosol, which further activates the NLRP3 or NLRC4 inflammasome complex; (2) ASM generated membrane rafts facilitate the formation of ROS via the activation of NADPH oxidases or mitochondrion, which are responsible for downstream inflammasome activation. The increase of cytokines, for instance IL-1β or TNF-α, is involved in TGF-β1 activation, cell apoptosis, collagen and elastin formation and finally lead to tissue injury and fibrosis.

The properties of tricyclic antidepressants and analogs lead them trapped and accumulated into the lysosome thereby detaching the ASM for lysosomal degradation, which makes these drugs as functional ASM inhibitor (Beckmann et al., 2014). The application of amitriptyline (Ma et al., 2017; Hong et al., 2019), desipramine (Chung et al., 2017), or imipramine (Biswas et al., 2017) normalizes ceramide levels and attenuates tissue injury and fibrosis. Importantly, a clinical trial shows systematic application of amitriptyline to CF patient results in beneficial effects on the lung and an increase in lung function (Nahrlich et al., 2013). Tricyclic antidepressants alone or in combination of other drugs may be novel, safe and effective candidate agents to reduce inflammation and prevent fibrosis. Notably, employing new genomic editing technologies, such as CRISPR/Cas9 to reduce ceramide accumulation may provide an exciting avenue to attenuate or delay the onset of inflammasome-associated diseases (Hong et al., 2019).

In the past 10 years, our knowledge has significantly increased in ASM and inflammasome signaling while new questions are raised. For example, what are the contributory roles of ASM in upstream mechanisms of inflammasome activating, and the structural basis of ceramide for signal recognition and complex assembly in inflammasome signaling? Does ASM regulate NLRP1, NLRC4, AIM2, NLRP6 or other inflammasome complexes? How do pathogens use ASM-inflammasome signaling to escape innate immune killing to enter the cells and activate caspases? How does ASM-regulated mitochondrial ROS interact with the inflammasome? How will this newly obtained knowledge be translated into other cells, tissue injury and fibrosis? Of note, the inflammasome is required for clearance of infective microorganisms and particularly for sensing sterile inflammation in tissue injury. Thus, a better insight into the balance in ASM regulating inflammasome signaling needs further determination.

Overall, this review provides an advanced understanding of the mechanisms in crosstalk of ASM with the inflammasomes, and defined their roles in infectious, metabolic, inflammatory, renal, and neurologic diseases. Our knowledge of ASM-ceramide system and inflammasome interaction would pave the way for further exploration of novel diagnostic, preventative, and therapeutic targets against tissue injury and fibrosis.

## Author Contributions

CL conceived and wrote the manuscript and generated the figure. All authors listed have made a substantial, direct and intellectual contribution to the work, and approved it for publication.

## Conflict of Interest

The authors declare that the research was conducted in the absence of any commercial or financial relationships that could be construed as a potential conflict of interest.
